# RISK FACTORS OF DISABILITY AMONG COMMUNITY-DWELLING OLDER ADULTS WITH HYPERTENSION IN THAILAND: THE HART STUDY

**DOI:** 10.1093/geroni/igac059.2128

**Published:** 2022-12-20

**Authors:** Utoomporn Wongsin, Tuo-Yu Chen

**Affiliations:** Taipei Medical University, Taipei City, Taipei, Taiwan (Republic of China); Taipei Medical University, Taipei City, Taipei, Taiwan (Republic of China)

## Abstract

About 60% of the older adults in Thailand experience hypertension, a condition that increases the risks of disability. However, little to no study has investigated modifiable risk factors to prevent disability in this segment of population in Thailand. Therefore, this study aims to investigate factors associated with disability among community-dwelling older adults aged 60 years or above with hypertension in Thailand. Longitudinal data (wave-1 [2015] and wave-2 [2017]) were from the Health, Aging, and Retirement in Thailand (HART) survey. Descriptive analysis and logistic regression analysis were employed to analyze the data. Outcome variables were difficulty with activity of daily living, hearing-related disability, vision-related disability, and pain-related disability in wave-2. Sociodemographic information, health behaviors/health status, mental illness, chronic diseases, and disability at baseline were independent variables. The results showed that older age significantly predicted difficulty with activity of daily living and hearing-related disability among community-dwelling older adults with hypertension in Thailand. Drinking alcohol significantly predicted vision-related disability. No factors were found to be a significant factor for pain-related disability. Our analysis provided useful information for the prevention of disability among older adults with hypertension in Thailand.

